# Modeling the Effects of Perceptual Load: Saliency, Competitive Interactions, and Top-Down Biases

**DOI:** 10.3389/fpsyg.2016.00001

**Published:** 2016-01-26

**Authors:** Kleanthis Neokleous, Andria Shimi, Marios N. Avraamides

**Affiliations:** ^1^Department of Psychology, University of CyprusNicosia, Cyprus; ^2^Department of Computer Science, University of CyprusNicosia, Cyprus; ^3^Department of Experimental Psychology, University of OxfordOxford, UK; ^4^Center for Applied Neuroscience, University of CyprusNicosia, Cyprus

**Keywords:** perceptual load, selective attention, distractor interference, dilution

## Abstract

A computational model of visual selective attention has been implemented to account for experimental findings on the Perceptual Load Theory (PLT) of attention. The model was designed based on existing neurophysiological findings on attentional processes with the objective to offer an explicit and biologically plausible formulation of PLT. Simulation results verified that the proposed model is capable of capturing the basic pattern of results that support the PLT as well as findings that are considered contradictory to the theory. Importantly, the model is able to reproduce the behavioral results from a dilution experiment, providing thus a way to reconcile PLT with the competing Dilution account. Overall, the model presents a novel account for explaining PLT effects on the basis of the low-level competitive interactions among neurons that represent visual input and the top-down signals that modulate neural activity. The implications of the model concerning the debate on the locus of selective attention as well as the origins of distractor interference in visual displays of varying load are discussed.

## Introduction

Our successful daily cognitive functioning relies heavily on our ability to select and process only a small subset of the information that is registered by our sensory organs and ignore the rest. The cognitive mechanism responsible for this is known as *selective attention* and has been studied vastly using various methodologies including behavioral experiments, electrophysiological, and neuroimaging studies, and computational modeling.

A central topic of debate in the field of selective attention has been the locus of attentional filtering within the stream of information processing. On one hand, “early selection” theories of attention (e.g., Broadbent, 1958) placed selection of visual stimuli at an early stage of processing claiming that it takes place on the basis of physical characteristics of stimuli such as their color, orientation etc., and prior to extracting their meaning. On the other hand, “late selection” theories (e.g., Deutsch and Deutsch, 1963) posited that selection occurs at a later stage and after all stimuli have been processed semantically. In between, the “attenuation” theory (e.g., Treisman, 1960) proposed that the selection process is the outcome of a filtering mechanism that attenuates rather than completely blocks the unattended stimuli whose threshold of activation is lowered based on significance, conditional probability, or contextual constraint. It is now accepted that the locus of selection is flexible (Chun and Wolfe, 2000; Luck and Hillyard, 2000; Luck and Vecera, 2002) and promising theories, such as the Perceptual Load theory (PLT) have been proposed to reconcile the debate concerning the locus of selection (in which we return to shortly).

Although PLT provides a sound theoretical proposal for determining when selection occurs and whether irrelevant stimuli are processed, yet a number of conflicting findings and alternative interpretations have been put forward. Perhaps the conflict arises because, in their majority, studies from both arenas have focused on studying perceptual load in the narrow context of perception (but see Lavie, 2010 for a review of how different cognitive processes may result in differential effects of perceptual load). Advances across the cognitive neurosciences suggest that understanding cognition is best achieved by treating perception, attention, and memory as well as their underlying neural circuits as part of an interacting network (Shapiro and Miller, 2011). Here, we present a computational model that aims to offer a unifying account for a set of experimental data that support or contradict PLT by taking into account the interactive relation of selective attention with perceptual and semantic competition and working memory.

Lavie and colleagues proposed PLT as a hybrid model that reconciles the long-standing debate concerning the locus of selection (Lavie and Tsal, 1994; Lavie, 1995; Lavie and Fox, 2000). According to this theory, the selection of stimuli may take place early or late depending on the perceptual load of the visual scene. This idea is based on the notion that attention is a pool of limited resources engaged by attentional tasks based on the processing demands they entail (see Eriksen and Eriksen, 1974, for a similar early account). For example, tasks of low perceptual load consume only minimal resources allowing spare resources to spill over to the processing of irrelevant items in the visual field; selection in this case is considered late. In contrast, high-load tasks consume all available attentional resources leaving none for the processing of irrelevant items. Selection, in this case, takes place early.

Perceptual load has been manipulated systematically and studies using a variety of dependent measures, including distractor interference (Lavie and Cox, 1997), negative priming (Lavie and Fox, 2000), and inattentional blindness (Cartwright-Finch and Lavie, 2007), have provided empirical support to the theory. For example, Lavie (1995) manipulated perceptual load by means of a difficult (high load) or an easy (low load) visual search with distractor interference being the dependent measure. In the high load condition participants searched for the letters “x” and “z” among six similarly-shaped letters arranged in a row while in the low-load condition they were presented with only the target in one of six possible locations in the row. In both conditions, a distractor letter—that participants were asked to ignore—was also presented above or below the row of letters. Depending on the condition, the distractor was compatible with the target (i.e., the same letter as the target), incompatible (i.e., the other target), or neutral (i.e., the letter “P”). Results from this experiment revealed that in the low-load condition, visual search was slowed down by the incompatible distractor compared to either the compatible or neutral distractor. Importantly, no differences between the three compatibility conditions were observed in the overall slower high-load task. These results suggested that participants were able to ignore the irrelevant distractor in the demanding high-load condition but not in the easy low-load condition. This finding is in line with Lavie's claim that early selection is “…impossible to achieve when the (attentional) capacity is not exceeded” (Lavie, 1995, p. 492) and Eriksen and Eriksen's argument that “…unutilized capacity cannot be shut off and, if there are other letters or stimuli present, they will be processed simultaneously with the target” (Eriksen and Eriksen, 1974, p. 144).

While PLT bridges the gap between early and late selection, a number of studies evaluating the theory have provided findings which seem at odds with its predictions. For example, a study by Johnson et al. (2002) showed that under certain conditions, findings compatible with early selection can be obtained in situations of low load. In this study, participants carried out conjunctive or feature visual searches as in Lavie and Cox (1997). In one condition though, a 100% valid central cue (i.e., an arrow presented in the center of the display pointing to the location of the upcoming target) appeared before the array. Results revealed that the interference exerted by an incompatible distractor that was presented next to the array was reduced in the cued low-load condition compared to a control low-load condition with no cue. The same result was also found by Neokleous et al. (2009) in an experiment with a partially valid peripheral cue (i.e., an asterisk appearing at the location of the target in 80% of the trials). Such findings are incompatible with at least a strong version of PLT, which claims that early selection cannot be achieved in conditions of low load (Lavie, 1995).

The absence of distractor interference under conditions of low load was also documented in a study by Eltiti et al. (2005). In addition to perceptual load and distractor compatibility, Eltiti et al. (2005) manipulated whether the distractor was presented as an onset or an offset. In the first reported experiment, the target always appeared as an onset, thus target and distractors were defined with the same property in the onset but not in the offset condition. While PLT predicts no effect of whether a distractor is presented as onset or as offset, other proposals predict that the distractor presentation affects selective attention. Specifically, the *contingent orienting hypothesis* (Folk et al., 1992) predicts that when both target and distractor are onsets, the distractor will attract attention. According to this theory, when searching for targets, people establish a high-level “attentional control setting” that guides the capture of attention by the target. Thus, when a distractor is defined by the same critical property that defines the target, it will inevitably attract attention. Furthermore, Eltiti et al. (2005) hypothesized that perceptual load modulates distractor saliency. That is, a single distractor in a low-load condition may be more salient than a distractor in a high-low condition and, based on the contingent orienting hypothesis, should produce interference if presented as an onset. In support of this hypothesis, the results from one of their experiments showed a significant interference effect in the onset low-load condition but not in the offset low-load condition. This result shows that top-down factors such as expectancies interact with the effects of perceptual load (see also Theeuwes et al., 2004; Sy et al., 2014). Furthermore, in a subsequent experiment reported by Eltiti et al. (2005), the saliency of the target was increased by making it slightly larger in size than the other items in the array. When the distractor appeared as an onset, a distractor interference effect was present for both low- and high-load conditions. In contrast, when the distractor appeared as an offset, no interference was observed in either high- or low-load conditions. Based on these findings, Eltiti et al. (2005) argued that what determines selective attention is the relative saliency of distractors and not perceptual load *per se*. Based on the findings of Eltiti et al. (2005) one could hypothesize that the visual saliency of distractors influences target detection by moderating selective attention mechanisms that rely on inhibitory interactions among stimuli. Furthermore, the strength of inhibitory signals may depend on the saliency values of the visual stimuli. These hypotheses are central to the model we present here and are discussed in more detail in subsequent sections.

In addition to the empirical evidence against it, the PLT has been also criticized on theoretical grounds. For example, Torralbo and Beck (2008) argued that the theory is unsatisfying because it offers no clear definition of perceptual load, and because the concept of exhaustive capacity cannot be reconciled with what is known about brain mechanisms. Indeed, while the PLT adopts the resource metaphor of attention (Kahneman, 1973), the concept of a resource remains a hypothetical construct that has yet to be identified as a specific neural structure or process. Thus, by relying heavily on the vague notion of a limited resource, the specificity of PLT is to some extent compromised. Moreover, as Giesbrecht et al. (2014) point out, the vague definition of load is susceptible to circular reasoning and inconsistent labeling of conditions (e.g., labeling linguistic or sensory manipulations as manipulation of perceptual load; see also Benoni and Tsal, 2013 for a discussion).

Torralbo and Beck (2008) took a step toward a more concrete formulation of PLT by specifying how neuronal competition in visual areas can give rise to perceptual load effects. According to their proposal, perceptual load effects arise from the competitive interactions of neurons in the early and intermediate visual areas to represent stimuli in the visual field, as well as a biasing mechanism that resolves competition in order to focus attention on a stimulus (see also Desimone and Duncan, 1995). In two experiments, Torralbo and Beck showed that factors that are known to modulate low-level interactions (i.e., the spatial density of the array and the presentation of target and non-target items in different hemifields) influence the extent of interference exerted by an incompatible distractor. Thus, they showed that the effects produced by perceptual load manipulations can also be obtained by manipulating other factors that are known to modulate the extent of competitive interactions.

More recently, Benoni and Tsal (2010) proposed an account for perceptual load effects on the basis of dilution, that is the inhibition exerted from the neutral letters of the circular array toward the distractors (see also Tsal and Benoni, 2010; Neokleous et al., 2011; Wilson et al., 2011). The idea of dilution is based on an early study by Kahneman and Chajczyk (1983) that used a Stroop color-naming task and showed that when naming the color of a bar, interference from an incongruent color word was reduced by about half when an irrelevant non-color word was added to the display; a phenomenon that has been termed as Stroop dilution. According to the Dilution account, the distractors in the visual search tasks employed by Lavie and Cox (1997) are processed in both load conditions but distractor interference in the high-load condition is eliminated due to diluting effects exerted by the non-target letters of the search array toward the distractor. In a low-load condition, in which no other letters appear in the array (e.g., Lavie, 1995), the interference exerted by the distractor toward the target is greater. Benoni and Tsal (2010) hypothesized that if dilution in a low-load condition is increased (e.g., by presenting the non-target letters in a different color or at a different location from the target), then no distractor interference should be found (see Section Modeling Dilution Theory for details on the task and the design of the experiment). Indeed, results from several experiments revealed no distractor interference under high-dilution low-load conditions, suggesting that dilution, and not perceptual load, is the critical factor for the presence of interference (see also Tsal and Benoni, 2010). Notably, the Dilution Theory relies on the presence of competitive interactions among stimuli that appear in the visual field. However, it seems to limit the interactions (1) between the non-target letters of the search array and the distractor, and (2) between the distractor and the target. Importantly, although the Dilution Theory can account for the greater interference in low load conditions when load is manipulated by varying the set size (as in Lavie, 1995), it has difficulty explaining the reduced interference observed in a number of studies in which set size was controlled (e.g., Lavie and Cox, 1997; Sy et al., 2014). Thus, it seems possible that PLT still holds for situations in which perceptual load is manipulated by other means (e.g., visual similarity between the target and the flankers; see Sy et al., 2014 for a discussion).

In summary, although PLT offers an attractive way of resolving the debate on attentional selection, a number of contradictory findings and alternative explanations such as the Dilution Theory challenge its validity. Furthermore, the theory is rather vague as it provides no clear definition of what constitutes high and low perceptual load and even so, it needs to be supported by neural mechanisms. The goal of this paper is to present a more concrete implementation of PLT through a computational modeling approach and to investigate how various alternative explanations can be reconciled with the basic tenants of PLT. In contrast to verbal/conceptual theories, computational models are characterized by conceptual clarity and precision as they require detailed and exact specification of the processes and parameters in order to run as computer programs (Newell, 1990; Sun, 2008). Therefore, this approach provides the benefit of overcoming vague and ill-defined definitions that are often found in verbal/conceptual theories (e.g., what constitutes high and low perceptual load).

Indeed, previous computational implementations of visual attention provide more precise definitions for perceptual load. For example, the Neural Theory of Visual Attention (NTVA; Bundesen et al., 2005, 2011) posits that visual objects compete for representation in visual short-term memory on the basis of an activation value that represents a visual categorization of the form “object x has feature i.” This activation value is determined by two equations, the *rate of processing equation* that computes the strength of the sensory evidence that object x belongs to category i (i.e., the firing rate of neurons coding particular features), and the *weight equation* that defines the attentional weight of object x (i.e., the number of cortical neurons in which an object is represented) which influences the rate of processing. According to NTVA, factors the influence the rate of processing, change the load of a task (Giesbrecht et al., 2014). Like NTVA, the present model needs no definition of perceptual load. Instead, whether a task is categorized as high vs. low load is relative and requires an arbitrary decision on a continuous measure.

The proposed model examines whether perceptual load effects in visual search tasks may result from low-level competitive interactions among neurons representing visual input, and top-down signals that modulate neural activity. Thus, the model aims at providing a parsimonious account for perceptual load^1^ effects that takes into account the low-level saliency of stimuli (e.g., Eltiti et al., 2005), their diluting effects (e.g., Benoni and Tsal, 2010), and high-level cognitive control mechanisms (e.g., Folk et al., 1992). The model examines whether a task is susceptible to distractor interference by considering the saliency of visual stimuli, their competition for cortical representation, and the effects from top-down factors. Although the model is designed specifically to simulate findings on PLT, it is structured as a general model of visual selective attention that can be applied to other tasks.

## The model

### Overview

The model was inspired by a number of influential theoretical accounts, such as the Global Workspace Model (Dehaene et al., 2003), the Biased Competition framework (Desimone and Duncan, 1995), and Niebur and Koch's (1994) neuronal implementation of selective attention. These accounts have provided a comprehensive framework for how selective attention may function at the neural level. In addition, details on how neural activity can be modified by top-down signals were also adopted from NTVA (Bundesen et al., 2005). The NTVA has been used in the past to account for behavioral effects in attention and perception by simulating the firing rates of single cells in the primary visual cortex, providing thus a bridge between cognitive function and neurophysiology. Finally, top-down feedback modulations corresponding to spatial and visual information were implemented in the model in the form of “competition” in a similar manner as in the model proposed by Hamker (2004).

**Figure 1:**
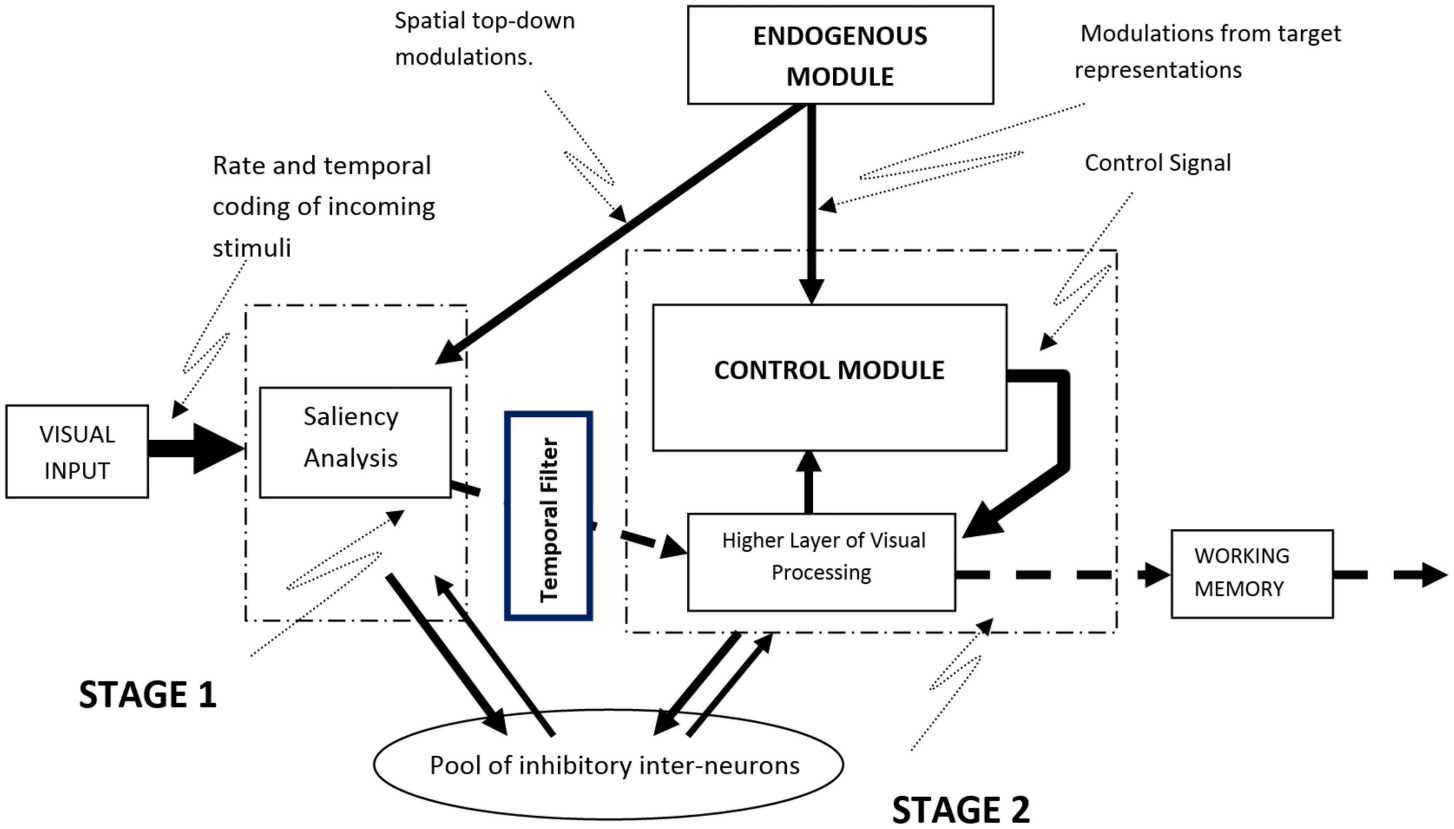
**A schematic overview of the computational model**. Visual input is subjected to saliency analysis whose output is used to establish the firing rates of neurons representing the stimuli. Competition among stimuli takes place in the model while top-down information from the Endogenous Module can bias the neural activity of stimuli that match the current goals.

The model is based on a system of dynamical equations implemented in the MATLAB/SIMULINK environment. The implementation combines the explicit specifications required by a connectionist spiking neural network model with background knowledge from Cognitive Psychology and Neuroscience. It simulates attention as a continuous stream of neural activity that is initially based on bottom-up visual information and top-down spatial attention signals but which gradually incorporates biases from goal-related information (i.e., the visual features of the target). Although processing in the model is continuous, for the sake of exposition it can be divided in two stages, implemented as spiking neural networks of Integrate-and-Fire (IF) and Coincidence Detector (CD) nodes. The first stage simulates the encoding of stimuli and the initial bottom-up competitive neural interactions among them, while the second stage involves top-down modulations of neural activity by target information (Figure 1). In the sections that follow we provide a general description of how neural activity is established and how it propagates through the model to activate a response. A detailed mathematical description of the elements of the model is provided in Appendix A of Supplementary Material.

### Stage 1: saliency and spatial attention

Every stimulus that appears in the visual field is represented in the model as a stream of spikes whose frequency is determined by the visual saliency of the stimulus and its location in the visual field. Saliency values are computed using the algorithm proposed by Koch and Ullman (1985) and implemented by Walther and Koch (2006; Saliency Toolbox—http://www.saliencytoolbox.net). Computing the saliency values for every location in the visual field produces a saliency map with values that represent the extent to which locations in the visual field may attract attention in a solely bottom-up manner (Zhaoping, 2002; Zhaoping and Dayan, 2006). These values are used in the model to establish the initial firing rates of the neurons corresponding to the locations of the various visual stimuli (see Appendix B in Supplementary Material for a detailed description of the algorithm used). Using a saliency analysis to establishing the firing rates of neurons allows stimuli that are more visually salient within the current context to be represented by spike trains with higher frequency. Once initial neural activity in the model is established, stimuli begin to inhibit the activity of neurons that correspond to other stimuli, by means of lateral and forward competitive interactions, in an effort to win the race to WM. As the frequency of neural activity is a direct function of visual saliency, the strength of the inhibition exerted by a given stimulus during the first stage of processing is determined by its saliency: highly salient stimuli exert stronger inhibition toward the representations of other stimuli in the visual field and are more likely to gain access to WM.

The importance of saliency for visual activity in the early stages of visual processing is supported by the finding that in the early areas of the visual cortex (e.g., area V1 and the FEF) a neuron's response can be significantly suppressed by contextual inputs that lie outside but near its receptive field (Nothdurft et al., 1999; Wachtler et al., 2003; Shibata et al., 2008). Furthermore, neurophysiological findings have documented that when two or more stimuli fall within the receptive fields of the same or nearby cells, competition for neural representation in visual areas V1 and V2 is initiated (Chelazzi et al., 1993; Reynolds et al., 1999; Reynolds and Chelazzi, 2004).

In addition to visual saliency, the model allows for top-down spatial signals to modulate the neural representations of stimuli that appear at specific locations. In experimental paradigms, these signals originate from the presentation of spatial cues that prime locations ahead of stimulus onset and from task instructions that guide attention toward specific locations or areas in the visual field. To simulate such top-down spatial interactions the model relies on spike trains held in a separate module, named the Endogenous Module, that represent specific task-relevant goals (e.g., direct attention to the location primed by a cue). In the case of a spatial cue, these spike trains enhance the firing rate of the neurons whose receptive fields coincide with the cued location. As a result, the neural representation of a stimulus appearing at that location will be amplified and in turn exert more inhibition toward visual stimuli that appear at other locations. Thus, the model considers the effects of top-down spatial factors on neural activity to be additive to those of visual saliency (see Appendix C in Supplementary Material for more information on how spatial attention is implemented in the model).

This implementation is in line with findings from several studies documenting that cues priming locations in the visual field increase the neural activity of neurons before and after the onset of stimuli (e.g., Gandhi et al., 1999; Silver et al., 2007; Shibata et al., 2008). It should be noted that despite the evidence for the early modulation of neural activity by cues, a number of studies have provided contradictory results. For example, in a study that involved recordings from the macaque V4, Sundberg et al. (2009) have shown that although spatial attention modulates center-surround interactions, the effects are relatively weak and do not provide a good basis for a strong spatial selection. However, in the model we consider spatial modulations at their initial state and we let the model incorporate these effects in the computations performed while neural activity progresses in the pathway defined by the model. Our results indicate that even when spatial modulations appear to be relatively weak initially, they can still have significant impact on the output.

### Stage 2: top-down modulations from target representations

The second stage in the model simulates the effect of goal-relevant information about the target (e.g., find letter N) on the neural activity that underlies the representation of the visual stimuli. These effects are implemented in the model in a way that produces both rate amplification (i.e., increase of spike firing frequency) and neural synchronization (i.e., more synchronized firing rates across neurons) in the representation of visual stimuli that match the target. This in line with neurophysiological evidence showing that attending a stimulus enhances the firing rate of neurons linked to the stimulus while at the same time it causes these neurons to fire in a more synchronous rhythm (e.g., Fries et al., 2001). Overall, the second stage in the model may reflect the interaction between the higher areas of the visual cortex and a fronto-parietal network responsible for maintaining goal-directed activity (e.g., Corbetta and Shulman, 2002; Posner and Rothbart, 2007), although it is widely accepted that even early visual areas remain involved in later stages of processing (Curtis and D'Esposito, 2003; Linden, 2007; Kelley and Lavie, 2011; Ioannides and Poghosyan, 2012; Sreenivasan et al., 2014).

In the model, neural synchronization is achieved by comparing the temporal structure of spike trains that represent the visual stimuli to the templates that contain the features of targets in the Endogenous Module. If resemblance above a pre-defined threshold is detected, then a *temporal filter* tunes the spike train of the visual input so that the timing of individual spikes becomes even more similar to that of the target. Notably, while the temporal filter alters the timing of spikes representing visual input, the average firing rate in the visual spike train remains unchanged. A result of this process is that the timing of spikes in the neural activity of visually relevant stimuli (i.e., stimuli that have common visual features with a target) becomes more similar both across these stimuli and with the target. This models synchronization that can take place both within a visual area (e.g., area V4; Fries et al., 2001) and across different areas in the brain (e.g., frontal eye field and V4; Gregoriou et al., 2009). More detail on the implementation of the temporal filter is provided in Appendix D of Supplementary Material.

The temporal filter mechanism used in the model was inspired by Crick and Koch's (1990) conjecture according to which the selection of stimuli can be made on the basis of synchrony across neurons. Based on neurophysiological findings that visual stimuli can elicit synchronized activity in the visual cortex, Crick and Koch (1990) suggested that a prerequisite for the presence of neural synchronization during attention tasks might be the appearance of synchronous impulses in selected neuronal populations. They proposed that visual selective attention causes changes to the temporal structure of the neural spike trains that represent the information to be selected, facilitating thus the transfer of the encoded information to WM. In a comprehensive review, Womelsdorf and Fries (2007) presented evidence from many studies showing that attention modulates the firing rates of neurons that represent the features of the attended stimulus causing synchronization.

Rate amplification in the model is implemented with a system that is referred to as the Control Module (see Appendix E in Supplementary Material for more detail). This module was inspired by the functional role of pyramidal cells in the visual cortex; pyramidal cells respond best to the coincident activation of multiple dendritic compartments (Spruston, 2008). In the model, a network of CD nodes evaluate the correlation between two streams of neural activity: neural activity that corresponds to visual stimuli and neural activity of the target representation. Based on the degree of correlation, a control signal is generated to amplify the neural activity of visual stimuli. The strength of the control signal depends on the total firing activity of the CD nodes in the Control Module. That is, if two signals are correlated, the CD nodes will fire more frequently, eliciting a stronger control signal.

In the Control Module, three nodes provide input to each CD node. Two of them are randomly selected from the total number of neurons corresponding to the neural activity of the visual stimulus and one from the target representation. The response of the CD nodes is explicitly dependent on the number of action potentials that arrive simultaneously at their inputs. In the current implementation at least two spikes must arrive synchronously for a CD node to fire and amplify the firing rate of the visual stimulus (Figure E1 in Appendix E of Supplementary Material). Thus, strong correlation between the neural activity of a visual stimulus and a target representation in the Endogenous Module will result in an increase of the firing rate, causing at the same time a gradual increase of synchronous firing by CD nodes.

Although the Control Module was not meant to model a specific brain region, its functions can be likely placed at the prefrontal cortex, in which previous studies have observed spontaneous activity correlated with both top-down and bottom-up processing (e.g., Fox et al., 2006), or at visual area V4 in which several studies have identified activity indexing the interaction between sensory information and the behavioral context (e.g., Reynolds and Desimone, 2003; Treue, 2003; Ogawa and Komatsu, 2004).

Following the top-down modulation of neural activity during the competitive interactions among stimuli in the second stage of processing, the visual stimulus that dominates the competition enters WM. The model includes a very simplistic WM network comprised of two nodes (Figure A1, Equations A11 and A13—Appendix A in Supplementary Material) that output a signal to mark the perceptual awareness of a visual stimulus when neural activity is sufficiently high to activate the WM nodes. Although this implementation is not meant to be a realistic model of WM, it allows us to obtain both accuracy and latency measures in order to simulate behavioral data. An accurate response is recorded when the neural activity of the correct stimulus forces the second working memory node to fire an action potential. The latency of this response signifies the time when the stimulus has entered awareness.

## Simulations

The model was used to simulate the pattern of results from 4 experiments in the perceptual load literature. For each of the conditions in the described experiment, 50 simulated trials were run with the model^2^. Median latencies are reported in all simulations. Except when noted, the parameters of the model were held constant across all data sets.

### Modeling the basic perceptual load pattern (Lavie and Cox, 1997)

We have opted for modeling the task used by Lavie and Cox (1997) which manipulated load by varying the similarity of the target and the flanking letters as opposed to that of Lavie (1995) which manipulated load by varying the set size of the display. This choice was based on the fact that although alternative explanations such as the Dilution Theory (Benoni and Tsal, 2010) can easily account for the findings in load tasks with set size manipulations, they have more difficulty with tasks in which set size is controlled (as in Lavie and Cox, 1997). In the study of Lavie and Cox (1997) participants searched for the letters X and N among either five similarly-shaped letters (high load) or five instances of the letter “O” (low load). The target and the flanking letters were arranged as a circular array. A larger distractor letter that participants were asked to ignore, was simultaneously presented to the left or to the right of the array. Depending on the condition, the distractor was compatible with the target, incompatible, or neutral.

**Figure 2:**
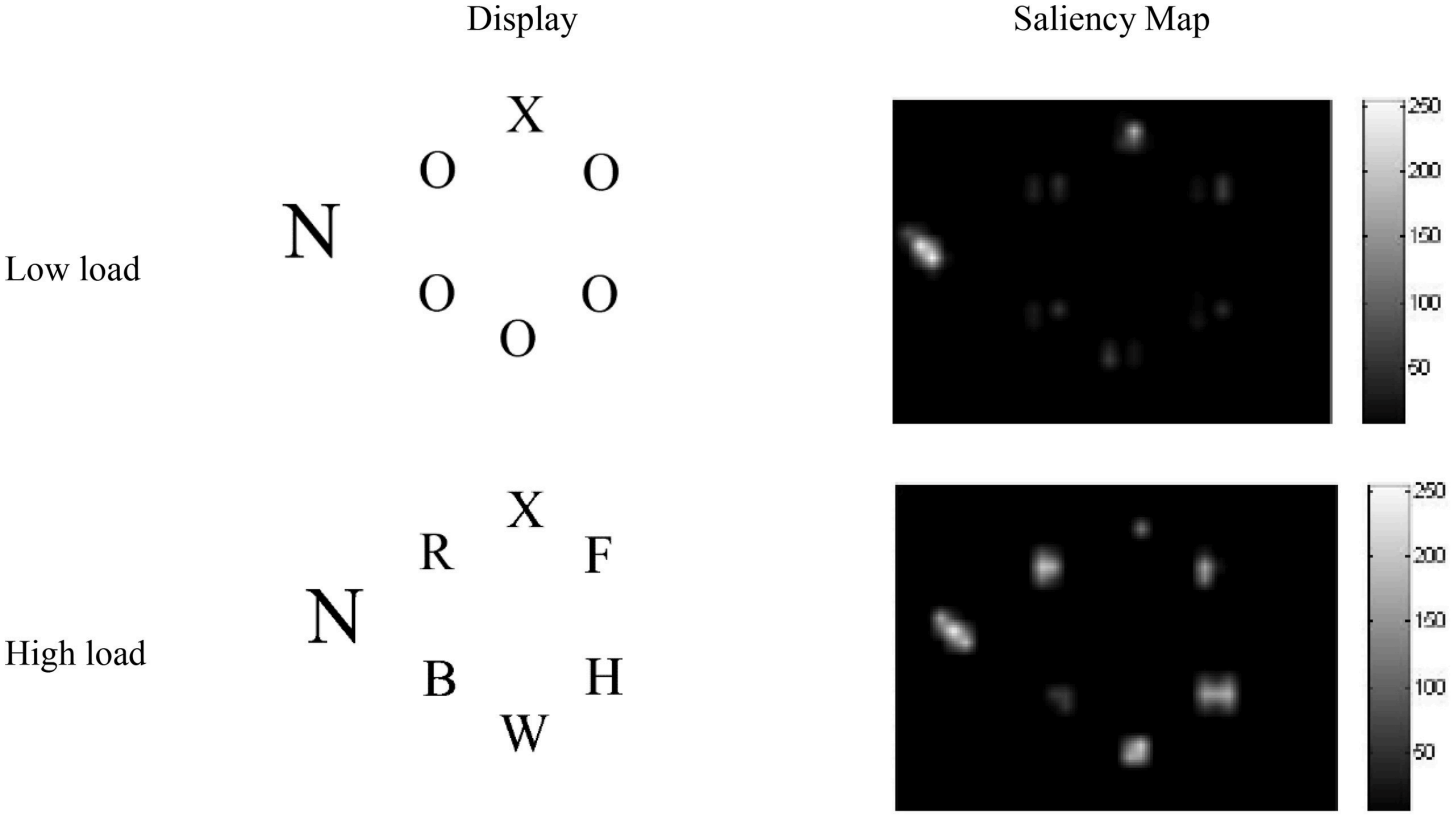
**Saliency analysis of high- and low-load displays**. In both conditions the larger distractor is the most salient stimulus. In the high-, but not the low-load condition, the flanking letters in the circular array are also highly salient.

For the simulations, screenshots from example displays used in a PLT experiment we conducted (Neokleous et al., 2009) were subjected to saliency analysis using the saliency toolbox of Walther and Koch (2006). The resulting saliency values were used to set the initial firing rates of the input neurons corresponding to each stimulus in the display, according to Equation B1 in Appendix B of Supplementary Material. An important result was that, in the low-load condition, the analysis yielded higher saliency values for the target than for the five O's in the array (Figure 2). This is because the identical non-target search letters that differed in shape from the target formed a unified background that caused the target letter (X or N) to become more salient and pop out of the display. In contrast, as seen in Figure 2, in the high-load condition other letters appearing in the circular array yielded high saliency values. However, in both load conditions, the distractor was more salient due to its larger size and was therefore associated with a spike train with a higher initial firing rate compared to all other stimuli in the display.

In addition to saliency, in order to establish the initial firing rates of neurons we took into account the fact that task instructions asked participants to focus on the stimuli presented within the circular search array and ignore the distractor. Therefore, top-down spatial signals with frequency of spikes equal to *f/N* (where N is the number of stimuli in the search array) were allowed to interact with the corresponding input neurons of the *N* stimuli as explained in Appendix C of Supplementary Material (in this case *N* = 6; 5 flankers and 1 target). The result of this interaction was an enhancement in the firing rate of neurons representing stimuli in the search array, which included the target.

As neural activity progressed to the second stage of the model, target-related information was allowed to exert an influence on neural activity. For example, the firing rates of neurons representing the targets X and N, which resemble the target templates in the Endogenous Module, were amplified. The degree of amplification depended on the level of similarity between the incoming stimuli and the target representations.

Throughout the progression of neural activity, stimuli competed for representation inhibiting each other in order to win the race to WM. In the high-load condition most stimuli that appeared in the search array had similar firing rates as they yielded about the same levels of saliency and they all fell within the circular area primed by task instructions. The distractor initially exhibited higher saliency due to its size but as it was presented outside the circular array, it received no spatial bias. As a result, its activation was about the same with that of other stimuli. The similar firing rates across stimuli in the display led to strong inhibitory interactions among all stimuli. Because of this, neural activity took longer to reach WM (i.e., to activate the first node of the WM network). Also, as the neural activity of the target was suppressed by other stimuli, the frequency of the synaptic input to the first WM node was low. As a consequence, the initiation of a response by the second node of the WM network was delayed until the threshold of perceptual awareness was reached through recurrent processing (see Appendix A in Supplementary Material for more detail on the functioning of the WM network). Furthermore, the strong combined inhibition exerted by the stimuli in the circular array toward the distractor suppressed its neural activity in the first stage of processing. The distractor could not influence performance even when it contained features that matched those in the target templates, and therefore, no substantial facilitation or interference was exerted by the distractor, regardless of whether it was compatible or incompatible to the target.

Due to iso-feature suppression (i.e., the suppression of neuronal responses when similar features are present in close proximity; Knierim and van Essen, 1992; Jones et al., 2001; Wachtler et al., 2003), the target was more salient than the surrounding O's in the low-load condition. In addition, the spatial top-down interactions in the circular area elicited by task instructions further amplified the neural activity of the target and the non-target letters in the search array. However, due to its higher activation the target exerted strong inhibition toward both the non-target flankers and the distractor. Compared to the high-load condition, there was smaller combined inhibition exerted from the stimuli of the search array toward the distractor, which also had high saliency due to its larger size. Therefore, the neural activity of the distractor was only suppressed to a small extent in the first stage of processing. In the second stage of processing, this activation was further increased in the case of an incompatible distractor whose features appeared in the target representations. The incompatible distractor generated strong inhibitory signals toward the target.

**Figure 3:**
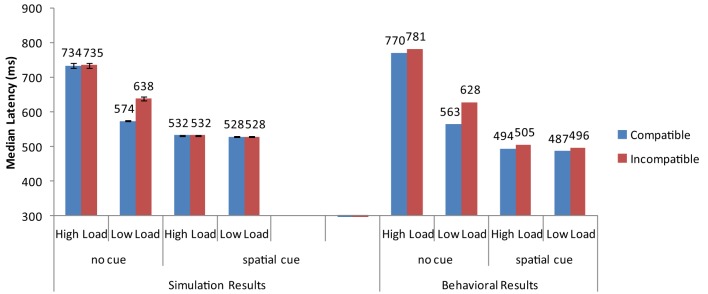
**Simulation results for the basic Perceptual Load pattern (no cue) and the cueing conditions of Johnson et al. (2002)**. Error bars represent standard deviations. Experimental data are depicted for comparison purposes. As no means for each combination of conditions are reported in Lavie and Cox (1997), the experimental data for the basic PLT pattern are taken from the replication of Johnson et al. (2002).

**Figure 4:**
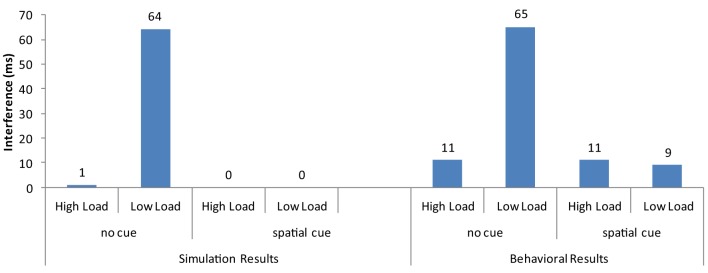
**Distractor interference (latency for incompatible minus the latency for compatible trials) from the current simulations and the behavioral results of Johnson et al. (2002)**.

Overall, the strong neural activation of the target resulted in shorter response latency than in the high-load condition (Figure 3) and the strong activation of the distractor produced interference in the incompatible condition (Figure 4). These are the two basic findings reported by Lavie and Cox (1997) in support of the PLT.

### Modeling the effects of spatial cueing on perceptual load (Johnson et al., 2002)

As shown in the previous section, the model was able to reproduce the basic pattern of perceptual load effects. A bigger challenge, however, was to examine whether the same model could also account for findings that are typically regarded as evidence against the PLT. As pointed out in the Introduction, one of these findings is that cueing can reduce distractor interference even in low-load conditions.

Johnson et al. (2002) examined how cueing interacts with perceptual load effects by adding to the basic conditions of Lavie and Cox (1997) high- and low-load conditions in which the target location was primed by a central symbolic cue. In these conditions, an arrow was presented in the center of the screen ahead of the onset of the circular array and pointed to the target with 100% validity. Results indicated that the cue (1) decreased reaction time in the high-load condition, and (2) eliminated the interference of the incompatible distractor in the low-load condition. The latter finding is problematic for the PLT as it demonstrates a circumstance where no interference is observed despite the low load.

A possible explanation for this finding is that the cue narrows attention to the potential location of the upcoming target, minimizing thus attentional leakage toward the non-target items and the distractor (Yantis and Johnson, 1990). Our model describes a way in which such narrowing of attention can take place in the brain. Specifically, we propose that a central cue that moves the focus of attention to a specific location in the display increases the firing rates of neurons whose receptive fields correspond to that location. This in line with findings that the spontaneous firing rates of V2, V3a, V4 (Freiwald and Kanwisher, 2004) and V1 neurons (Kastner et al., 1999), whose receptive fields correspond to cued locations, increase even before a stimulus is presented at that location. This attentional modulation of spontaneous activity takes place regardless of the identity of the stimulus (i.e., target vs. distractor) that will later occupy the cued location. As this enhanced spontaneous activity is observed in response to cues and instructions to orient attention to particular locations, it can be best thought of as reflecting the effects of attentional control on stimulus processing (Hopfinger et al., 2004; see Raftopoulos, 2009 p. 67–69 for further discussion). While the attentional modulation of spontaneous activity following a valid cue provides an advantage for processing the target, it still allows all items to be encoded and processed for meaning. In the model, we simulated the effects of a 100% predictive cue by adding a top-down spatial signal (i.e., focus attention on the item in the cued location) in the Endogenous Module to amplify the firing rate of the neurons that correspond to the target location. As explained in Appendix C of Supplementary Material, the frequency of this signal was *f/N* = *f*, since a single spatial location was primed by the cue (i.e., *N* = 1).

The cue-related spatial top-down signal raised the neural activity of the target from the very early stages of processing. As a result, the target induced strong inhibitory signals toward the neurons that encoded the distractor and the non-target search array letters. This interaction reduced overall reaction time, particularly in the more difficult high-load condition. It also eliminated distractor interference in the low-load condition (Figure 3). Thus, by simply increasing the neural activity of the target to simulate the effects of cueing, the model was able to reproduce the pattern of findings reported by Johnson et al. (2002).

### Modeling the effects of a central distractor (Beck and Lavie, 2005)

Beck and Lavie (2005) conducted an experiment in which they manipulated, in addition to perceptual load, the location of the distractor. In the peripheral condition, the distractor appeared adjacent to the array as in other PLT studies (e.g., Lavie and Cox, 1997). In the central condition, the distractor was presented in the center of the display within the search array. The target was presented within a circular array of either small circles (low-load condition) or similarly-shaped letters (high-load condition). Results from the experiment showed that (1) interference from an incompatible distractor in the low-load condition was greater with the central than the peripheral distractor, and (2) distractor interference was observed in the high-load condition when the distractor was central but not when it was peripheral.

For the peripheral condition, we used the model as described in Section Modeling the Basic Perceptual Load Pattern (Lavie and Cox, 1997) where the simulations produced the same pattern of latencies obtained for the findings of Lavie and Cox (1997): interference from an incompatible distractor was present in the low-load condition but not in the high-load condition.

To simulate the central distractor conditions, we allowed spatial top-down signals to raise the initial firing rate of the distractor as its location was within the area primed by task instructions (i.e., search for the target in the circular array). Thus, we assumed that attention functions as a spotlight and cannot be allocated in a circular region that excludes its center (see Müller and Kleinschmidt, 2004).

**Figure 5:**
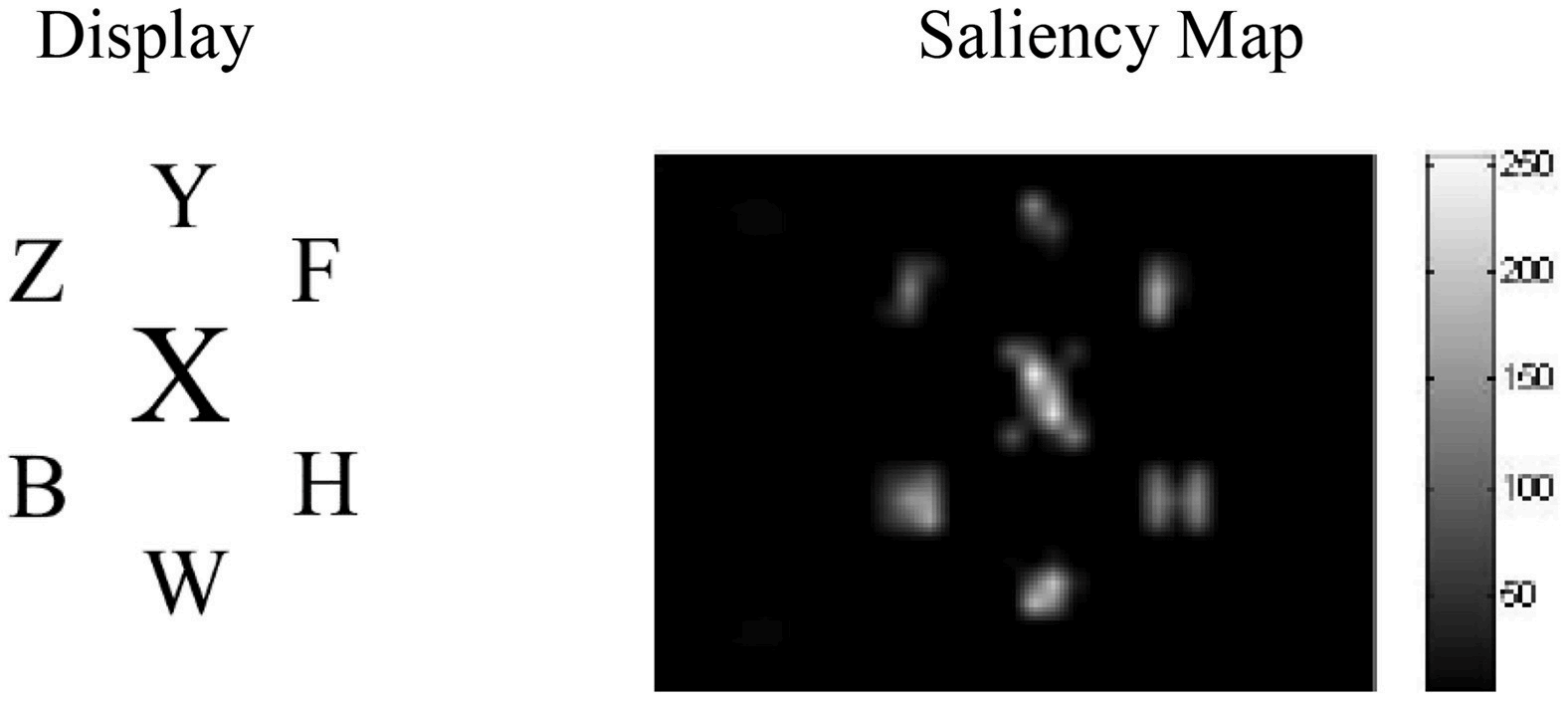
**The results of the saliency analysis for the high-load central distractor condition of Beck and Lavie (2005)**.

Figure 5 shows the results from the saliency analysis in the high-load central distractor condition. As seen, the bigger distractor is highly salient even before any spatial top-down effects.

**Figure 6:**
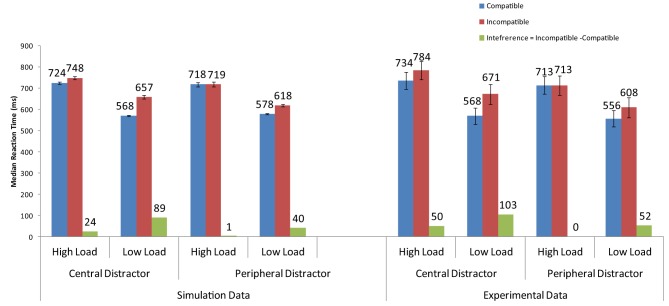
**Simulations of the findings of the experimental data of Beck and Lavie (2005) for central and peripheral distractor conditions**.

When the firing rate of the neurons corresponding to the letters in the circular array, this time including the distractor, are increased by the spatial bias, the distractor's neural activity becomes sufficiently high to cause interference when incompatible with the target (Figure 6). In addition, the simulations showed that distractor interference in the low-load condition is about twice as much when the distractor is central than peripheral. This result is also present in the findings of Beck and Lavie (2005).

### Modeling dilution theory

**Figure 7:**
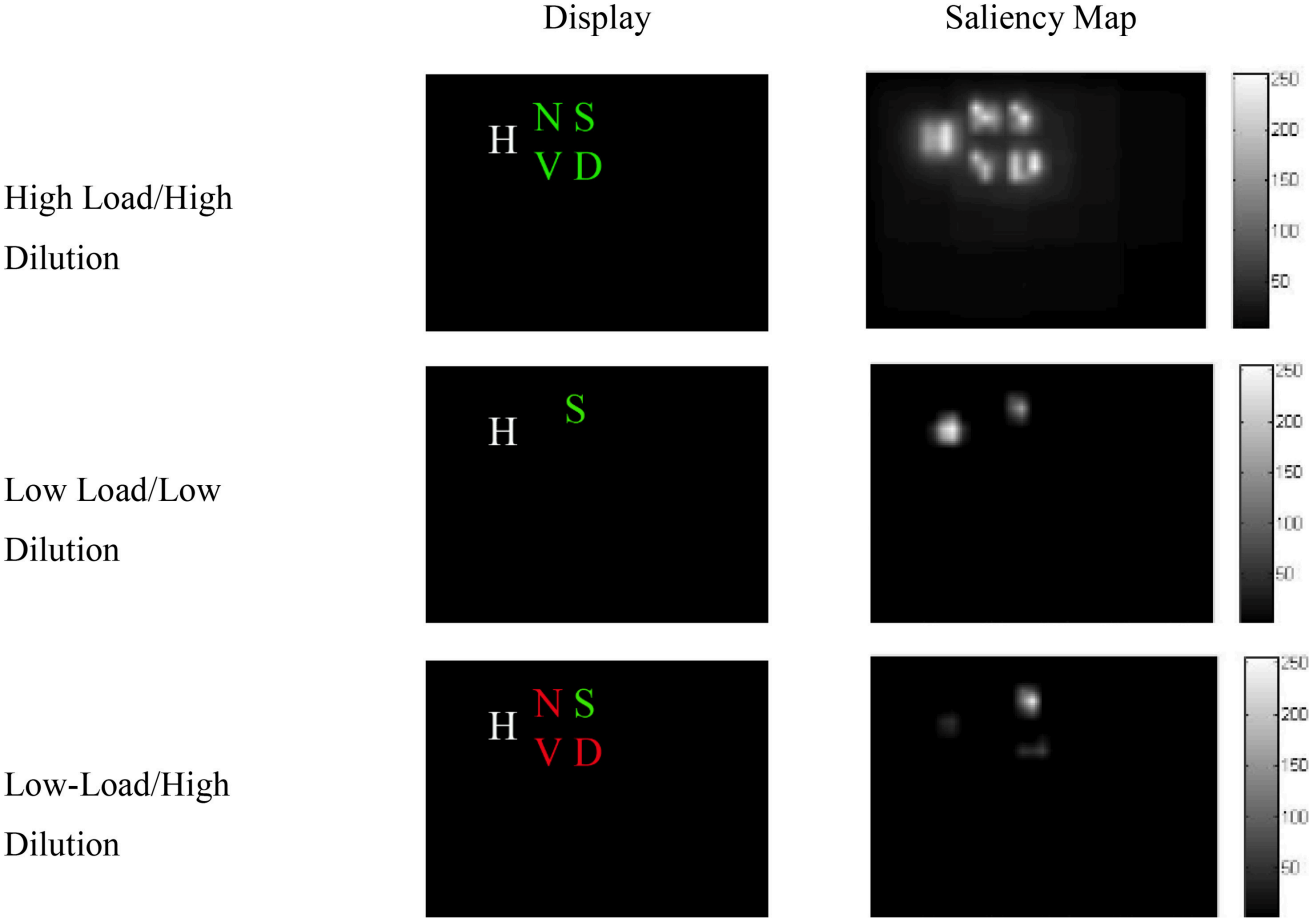
**Saliency analysis for the three conditions of Benoni and Tsal (2010)**.

Benoni and Tsal (2010) conducted a number of experiments to provide evidence that perceptual load effects arise from diluting effects exerted by the non-target letters of the search array toward the distractor. In their task, they asked participants to identify a target letter in three different conditions: low-load/low-dilution, high-load/high-dilution, and low-load/high-dilution. In the low-load/high-dilution condition, participants searched for a red target among three green non-target letters or a green target among three red letters. In both cases, the four letters were presented at the corners of an imaginary square and a larger white distractor appeared adjacent to the array. The high-load/high-dilution condition differed in that the four letters of the search array, including the target, were displayed in the same color, either red or green. Finally, in the low-load/low-dilution condition, the red or green target was presented without any accompanying non-target letters (see Figure 7).

Results showed that overall latencies were shorter for the low-load/low-dilution and the low-load/high-dilution than the high-load/high-dilution condition. Furthermore, latencies were equal for the low-load/low-dilution and the low-load/high-dilution conditions indicating that presenting the non-target letter in a different color from that of the distractor effectively reduced the difficulty of visual search. Importantly, however, distractor interference was present in the low-load/low-dilution condition but not in the low-load/high-dilution condition. The absence of interference in a low-load condition is problematic for PLT which claims that spare resources are automatically spilled over to distractor processing. Therefore, Benoni and Tsal (2010) argued that the effects reported in perceptual load tasks are caused by the varying levels of dilution that non-target letters exert on the distractor.

**Figure 8:**
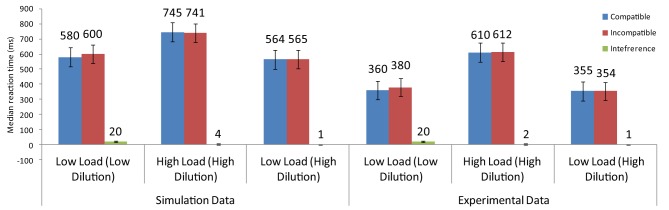
**Simulation results for the findings of Benoni and Tsal (2010)**.

The model that simulated the basic PLT findings [Section Modeling the Basic Perceptual Load Pattern (Lavie and Cox, 1997)] was applied to the conditions of Benoni and Tsal (2010). Notably, the saliency analysis produced higher values for the target than the non-target letters in the low-load/high-dilution than in the low-load/low-dilution condition, due to the fact that in the low-load/high-dilution the target was presented in a different color from that of the other stimuli. Thus, the neurons corresponding to the target location had in this condition high initial firing rates, allowing the target to exert strong inhibitory signals to all other stimuli. This minimized the effect that the distractor had on performance. As a result, replicating the behavioral results, the model produced distractor interference only in the low-load/low-dilution condition (Figure 8). Furthermore, it captured the overall slower latency observed in the experiment for the high-load/high-dilution condition compared to the other two conditions.

While the model reproduced the pattern of findings reported by Benoni and Tsal (2010), it did so in a way that differs somewhat from what the Dilution Theory claims. The Dilution Theory attributes perceptual load effects exclusively to inhibition exerted by non-target letters of the search array toward the distractor and assumes that the target is immune to inhibition. Our model does not need to make this assumption. Instead, it allows inhibitory signals among all stimuli in the display and does not differentiate between the target, the distractor, and the non-target letters. Finally, it should be noted that the same model can account for the findings of Benoni and Tsal (2010) that support the Dilution theory but also those of Lavie and Cox (1997) that the Dilution Theory has difficulty explaining.

## Discussion

The model was able to simulate the basic pattern of findings observed in perceptual load studies (e.g., Lavie and Cox, 1997), the findings regarding the interaction of perceptual load with cueing (Johnson et al., 2002; Neokleous et al., 2009), the findings from the comparison of central and peripheral distractors (Beck and Lavie, 2005), and the results supporting a dilution explanation (Benoni and Tsal, 2010). Notably, this was achieved by changing across simulation different data sets only the parameters of the model that were justified by relevant research. This explains why although the pattern of results were reproduced, in some datasets the actual simulation results differ in magnitude from those that resulted from the behavioral studies.

In summary, the simulations showed that the model accounts for the presence of distractor interference in low-load but not in high-load conditions when the distractor is presented in the periphery. Moreover, it can also account for the presence of interference from a central distractor even in the high-load condition. In addition, it can explain how distractor interference in low-load conditions can be eliminated by cueing.

Importantly, the present computational model offers an explicit and concrete account for perceptual load effects that does not rely on vague descriptions of perceptual load. In fact, the model does not rely on any definition of what constitutes high or low load. The terms retained in the description of the simulations simply as labels for the various conditions to allow the reader connect the simulation results with empirical findings from other studies. Instead, any visual search task of the type used in PLT studies could be subjected to the analysis described in the model. Here, we show that using the tasks in the form used in previous behavioral studies can lead to the pattern of interference reported by these studies.

The model attributes the effects of what has been considered Perceptual Load to the interactions between low-level saliency and top-down spatial and semantic goals. On one hand, the model includes continuous inhibitory interactions among the stimuli in the visual field whose relative saliency determines the strength of the inhibitions that are exerted. Thus, it posits that the extent of the diluting effects among stimuli is highly dependent on saliency. In addition, the model allows for top-down signals to bias this competition by amplifying the activity of neurons representing stimuli that match the spatial goals and the stored target representations. In other words, whether or not people are susceptible to distractor interference in a given task depends on the relative saliency of the stimuli in the visual field as well as their current goals. Therefore, the model is compatible with claims that saliency is an important determinant of perceptual load effects (Eltiti et al., 2005) and that the neural basis of perceptual load arises from competitive interactions in the visual cortex, along with biasing mechanisms for resolving these competitions in favor of the target (Torralbo and Beck, 2008). Since our model allows for modulating the activity of neurons that match the current goals, it can easily account for the effects of cueing (e.g., Johnson et al., 2002) though a simple amplification of the firing rates of neurons that correspond to cued locations.

Although the model does not discard PLT, it disagrees with it on *one* important aspect. PLT assumes that perceptual resources are allocated following a two-step procedure. That is, perceptual resources are *first* allocated to task-relevant items (e.g., to items that appear in positions where the target may appear) and only if there is remaining capacity it can then spill over automatically to task-irrelevant items. This is assumption is not retain in the proposed model. Our conjecture is that all items in the display are processed simultaneously in a single step and the probability of distractor interference is determined by the features of the stimuli and potential top-down biases. To that respect, our model agrees with NTVA, which also proposes a single-step allocation mechanism (see Giesbrecht et al., 2014 for a discussion). Importantly, the simultaneous allocation of resources to task-relevant and task-irrelevant items is supported by empirical findings. For example, in *one* study, Kyllingsbæk et al. (2011) had participants report the identity of four targets presented in a circular array six possible locations while ignoring neutral distractors presented in the periphery. The number of distractors (zero, one, or two) and their color (same as the targets or different) was also manipulated. If the allocation of resources takes place in *two* steps as assumed by PLT, then performance on reporting the targets should not be affected by the manipulations on the distractors. However, results shows that both the number of distractors and their color influenced performance. That is, fewer targets were correctly reported as the numbers of distractor increased and more targets were correctly reported when the distractors appeared in different vs. same color as the targets. These findings support the single step allocation procedure proposed by NTVA and the current model.

It should be noted that, although the current model was not designed so that its parts exhibit 1:1 correspondence to brain regions, several aspects of the model are compatible with what is currently known about attention and its underlying brain mechanisms.

First, the model can account for attentional capture, that is, the involuntary allocation of attention to stimuli on the basis of their salience. There is some debate in the literature on whether attentional capture is penetrable by top-down processes. On one hand, a number of studies using the additional-singleton paradigm suggest that irrelevant distractors capture attentional resources and slow down responses to the target even when distractors have no common defining properties with the target and therefore do not match the current goals (e.g., Theeuwes, 2004; Hickey et al., 2006). On the other hand, studies using the modified spatial cuing paradigm suggest that attentional capture is modulated by top-down attentional control settings, since cues can reduce response latencies to targets when they are defined by the same properties as the targets (e.g., Folk et al., 1992; Folk and Remington, 1998; Eimer and Kiss, 2008). Our model can account for these findings. While initially in the model neural activity depends on the saliency of visual stimuli, top-down factors are allowed to exert influence even on this early neural activity, in line with Folk et al.'s (1992) Contingent Attentional Capture hypothesis. The model allows for spatial top-down goals to modulate initial neural activity, consistent with studies showing that spatial cues may increase the response of neurons that correspond to cued locations even prior to the onset of a stimulus (e.g., Shibata et al., 2008). Moreover, the model allows for semantic top-down goals to influence neural activity at a later stage of processing that, nevertheless, precedes the onset of perceptual awareness. Thus, the model provides an explicit mechanism for how attentional capture can take place on the basis of saliency while allowing at the same time the influence of the top-down attentional set.

Second, by incorporating a low level saliency map, the model is sensitive to the effects of iso-feature suppression, that is the fact that the response to an input feature (e.g., orientation, color, or motion direction) is more suppressed when there are similar rather than different input features nearby (e.g., Zhaoping, 2002). The use of a saliency map to generate the initial firing rates of every incoming stimulus in the model provides an initial attentional advantage to certain stimuli that stand out in the context, as is the case of a target in a low-load condition.

Finally, the use of coincidence detector neurons in the model is consistent with a growing body of evidence that the synchronization of neural activity plays a role in perception and attention. For example, the timing activities in the posterior parietal cortex and an earlier area in the visual pathway (V4) were synchronized in macaques carrying out a visual matching task, as revealed by simultaneous neural recordings from the two regions (Saalmann et al., 2007). Attending a stimulus has been shown to involve enhanced oscillatory coupling between area V4 and the Frontal Eye Fields in the prefrontal cortex (Gregoriou et al., 2009). In line with these findings, the present model produces synchronization of neural activity across brain areas when the visual input matches the neural activity that maintains top-down information.

## Conclusion

Overall, the model presents a concrete and plausible way of how findings termed as perceptual load effects can arise in various experimental conditions. Importantly, the model not only simulates the basic pattern of findings that support PLT, but it can also account for findings that are considered contradictory to the theory. In contrast to PLT that posits that selective attention can be early or late depending on the perceptual demands of the task, the present model provides one way of accounting for perceptual load effects on the basis of late-selection mechanisms. Although the physical properties of stimuli influence their saliency and may provide an advantage for some stimuli solely on the basis of bottom-up processing, all stimuli are eventually subjected to higher-level processing in the second stage of processing of the model. This higher-level processing of each stimulus is deployed by the Control Module, which is responsible for assessing the match between encoded stimuli and the current goals. According to the model, all stimuli in a display, including unattended distractors, are encoded and identified (see Driver and Tipper, 1989 for a related proposal). Critically, this higher-level processing of the stimuli precedes perceptual awareness; in fact, even after such processing, some stimuli may never reach the threshold of perceptual awareness. This is compatible with findings that stimuli presented below the threshold of conscious awareness may still influence behavior (e.g., Naccache et al., 2002). Finally, it should be pointed out that the model requires no formal definition of what constitutes high and low perceptual load. Instead, according to the model, what determines the presence of the effects reported by Lavie (1995) and others (e.g., Benoni and Tsal, 2010) are the details in the low-level competitive interactions among neurons that represent visual input and the top-down signals that modulate neural activity.

In closing, it should be acknowledged that the model exhibits important limitations. For example, the WM network that is included for the sake of implementation is simplistic and, by no means, simulates the complex relation between attention and working memory (e.g., Shimi et al., 2015) or the possible interaction of working memory with perceptual load (e.g., de Fockert, 2013). Also, certain theoretical constructs in the model (e.g., the Control Module) cannot clearly mapped to brain structures, despite the evidence for their functioning. In light of these limitations, the model can be considered as a working theory for how distractor interference is produced in different tasks. Importantly, this theory combines existing explanations and hypotheses (e.g., saliency, dilution, attentional leakage, top-down attentional control) to synthesize a parsimonious account for perceptual load effects on the grounds of a biologically plausible neural network.

## Author contributions

KN and MA conceived and designed the research; KN implemented the computational model and carried out the simulations; KN, AS, and MA interpreted the simulation results; AS and MA drafted the introduction and discussion sections; KN, AS, and MA prepared the technical parts of the article, the appendices, and the figures.

## Funding

Funding for the conducted research was provided by grant 0308(BE)/16 from the Cyprus Research Promotion Foundation. The Center for Applied Neuroscience at the University of Cyprus provided partial funding for the required Public Access charge.

### Conflict of interest statement

The authors declare that the research was conducted in the absence of any commercial or financial relationships that could be construed as a potential conflict of interest.

## Footnotes

^1^The model requires no explicit definition of what constitutes high or low perceptual load. The terms are used as labels for conditions included in previous empirical studies to allow easy comparison across studies.

^2^The number of simulation runs was decided based on pilot data showing that a stable pattern of results could be obtained with these many runs, and would not change with additional runs. In fact, as the stochastic effects of the model are rather limited, a stable pattern of results could have been achieved with even a handful of trials. Following the logic of Ritter et al. (2011), we consider the model as a theory and we therefore give more emphasis on the general pattern of results produced than on the exact fit of data.
