# Supplementary material for: Modeling the Effects of Perceptual Load: Saliency, Competitive Interactions, and Top-Down Biases
Source: Front Psychol. 2016 Jan 26;7:1. doi: 10.3389/fpsyg.2016.00001 (PMC4726798; doi:10.3389/fpsyg.2016.00001)
Supplement: Supplementary file 1 [file DataSheet1.docx]

# Appendix A. Mathematical formulations of the model

# A.1. Integrate and fire nodes

The basic computational unit used in the model is the graded response neuron, defined by the membrane Eq.A1.

$\tau_{m}\frac{dV}{dt}=E_{L}-V(t)+R_{m}I_{s}(t)$ Eq.A1

***V*** is the membrane potential of each neuron, ***τm*** $\tau_{m}$is the membrane time constant, and ***EL*** $E_{L}$is the resting potential of the membrane. The membrane potential can be seen as a measure of the extent to which a node is excited. ***Is(t)*** r $I_{s}(t)$epresents the total synaptic current and is a simple combination of pre-synaptic excitation and bias currents that increase the membrane potential, with inhibition currents that reduce the membrane potential of the node. The total summation of the excitatory and inhibitory currents influences the actual membrane potential at each time instance. Finally, ***Rm***$R_{m}$ is the membrane resistance of the neuron. In brief, Eq.A1 determines how the membrane potential ***V*** of each neuron develops over time after an input current $I_{s}$ ***Is*** is applied. The value of the membrane potential increases until it reaches a specific threshold (***Vth***) at which a spike is emitted and ***V*** resets to its initial condition or resting potential ***Vres***$V_{\mathrm{reset}}$ . Subsequently, a refractory period of 2 ms is applied before the neuron model is allowed to integrate again any pre-synaptic currents.

The $I_{s}\left( t \right)$ term ***Is*** $I_{s}\left( t \right)$ in Eq.A1 quantifies the synaptic currents that are mediated by the excitatory receptors AMPA and NMDA (activated by glutamate, gAMPA and gNMDA) and the inhibitory receptor GABAA and GABAB, as shown in Eq. A2.

$I_{s}\left(t\right)=\left(I_{AMPA}\left(t\right)+I_{NMDA}\left(t\right)+I_{GABA_{A}}\left(t\right)+I_{GABA_{B}}\left(t\right)\right)$ Eq.A2

In the model, the synaptic inputs correspond to the sum of the excitatory and inhibitory synaptic currents (*Iexc(t)+ Iinh(t)*).

In the framework of the integrate-and-fire model, each pre-synaptic spike generates a post-synaptic current pulse that is driven towards the input of the following neuron as shown in Eq. A3

$I_{s}\left(t\right)=\left(I_{exc}\left(t\right)+I_{inh}\left(t\right)=g_{exc}\left(t\right)(Es_{exc}-V)+g_{inh}\left(t\right)(Es_{inh}-V)\right)$ Eq.A3

where

Eq.A4

$\bar{\mathbf{g}}$ in eq. A4 is the maximal excitatory or inhibitory conductance and *wexc, winh* refer to the excitatory and inhibitory synaptic weights.

*Ps(t)* ${P_{s}(t)}_{j}$ determines the synaptic conductivity and can be modeled by a simple exponential decay with time constant τs as shown in eq.A5.

$P_{s}=\frac{1}{e^{\frac{t}{\tau_{s}}}}+\frac{(\Theta(t-t_{k})e^{\frac{t_{k}}{\tau_{s}}})}{e^{\frac{t}{\tau_{s}}}}$ Eq.A5

In Eq.A5, Θ represents the Heaviside step function (zero for negative arguments, unity for zero or positive arguments, Eq. A6).

$\Theta(x)=\left\{\begin{matrix} 0 \\ 1 \end{matrix}\text{ }\begin{matrix} x<0 \\ x>=0 \end{matrix}\right.\text{ }$ Eq.A6

Finally, by considering that the synaptic conductivity is a function of the incoming pre-synaptic spikes (i.e $P_{s}\text{= }f (\delta(t-t_{k}))$), the total input current can be similarly modeled as a function of the synaptic weights and the spike train sequences of the pre-synaptic neurons (Eq.A7).

Eq.A7

As the coupling interactions in the model are performed in discrete time, the corresponding mathematical expressions are adjusted accordingly. Specifically, spike duration in biological neurons has a finite duration in the order of 1ms. Therefore, each spike in the model is represented by a pulse of unity amplitude and duration of 1ms, i.e., in the discretization processes each time step δt is taken to be 1ms.

In line with the above, the basic membrane potential differential equation (Eq. A1) for a neuron i and the mathematical expression of the synaptic currents after a formal time discretization are given by Eq.A8 and A9:

$V_{i}(t+\delta t)=(E_{L}-V(t))(1+\frac{\delta t}{\tau_{m}})+\frac{R_{m}}{\tau_{m}}I_{s}(t)\delta t$ Eq.A8

$Ps_{i}(t+\delta t)=\frac{Ps_{i}}{\tau_{s}}(1-\frac{\delta t}{\tau_{m}})+\sum_{k} \delta(t-t_{k})\delta t$ Eq.A9

# A.2. Coincidence detector (CD) nodes

Traditionally, CD neurons are modeled with a very short membrane time constant τm that can change rapidly. However, another way to model coincidence detection is based on a simple case in which separate inputs converge to a common target.

More precisely, if Ψ(t) is a binary row vector denoting the states of neuron A and B at time t and C(t + 1) the state of neuron C at t + 1, then the outcome of C at t +1 can be expressed as:

$C(t+1)=\Theta(\Psi(t)-\theta)$ Eq.A10

With $\Theta$ Θ being the Heaviside step function, and *θ* the specific threshold for a number of pre-synaptic spikes that are needed to arrive synchronously in order for the output neuron C to induce a spike.

# A.3. Working memory nodes

The working memory network in the model is comprised of two nodes with the membrane potential of the first evolving according to Eq. A11:

$V_{WM1}(t+\delta t)=(E_{L}-V_{WM1}(t))(1+\frac{\delta t}{\tau_{m}})+\frac{R_{m}}{\tau_{m}}I_{s}(t)΄\delta t$ Eq. A11

The total input synaptic current$\mathbf{I}_{\mathbf{s}}\left( \mathbf{t} \right)\mathbf{'}$ *Is(t)’*of the first WM1 node is developed based on the neural activity of the specific visual stimulus that the WM nodes corresponds to (e.g. for the letter Z or X). *Is(t)’*at each time instance is expressed by:

$I_{s}(t)΄=I_{s}(t)+I_{s}(t+1)$ Eq.A12

As seen in Eq.A12, the synaptic input of the WM­1 node is sustained and whenever a new synaptic input arrives it is added to the previous value. As a consequence, the response frequency of the WM1 node varies depending on the strength of the incoming neural activity.

In other words, high frequency of spike activity in the input of WM1 node, induces spike activity of high frequency in the output response of WM1 node. In contrast, low frequency input activity results in fewer spikes on the output of WM­1 node. Hence, the response of the second working memory node (WM­2) is explicitly dependent on the response of the WM1 node:

$V_{WM2}(t+\delta t)=(E_{L}-V_{WM2}(t))(1+\frac{\delta t}{\tau_{m}})+\frac{R_{m}}{\tau_{m}}I_{WM1_{s}}(t)\delta t$ Eq.A13

Within the working memory network framework, important inhibitory interactions are also considered. Specifically, the output of WM­1 node is used for inhibition between other WM1 nodes that correspond to competitive stimuli that might appear in a different spatial location of the visual field (for example as is the case of incompatible stimuli).

Once the second working memory node (WM2) fires an action potential, perceptual awareness of the stimulus that corresponds to the specific WM module is assumed. The system’s response time is taken to be the time that WM2 node fires an action potential (see also figure A1).


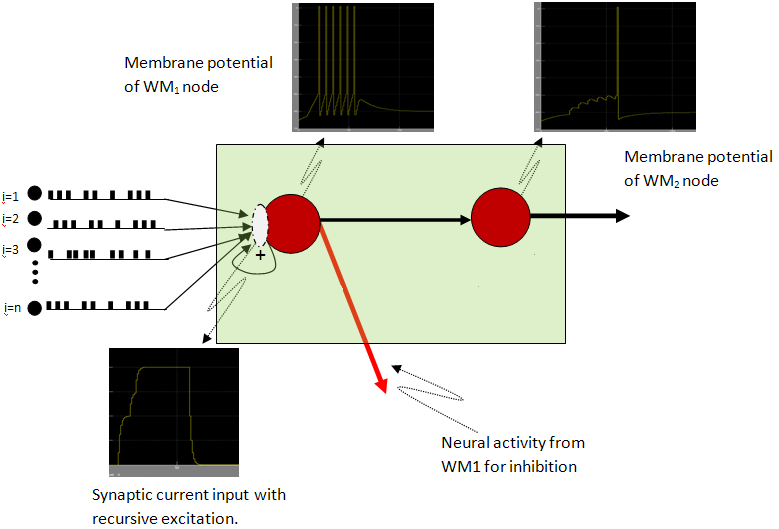


*Figure A1*. Schematic depiction of the Working Memory Module.

**Appendix B**

**Establishing initial neural activity based on saliency**

Each stimulus in the visual field is encoded through 12 input neurons whose receptive fields are associated with the spatial positions at which the stimuli appear. These neurons encode stimuli as spike trains, i.e., series of discrete action potentials that are represented in the model as binary events (0’s and 1’s) denoting the absence or presence of a spike/action potential.  For example, if the firing rate of an input neuron A is FRA, then the corresponding spike train can be seen as a Bernoulli process with probability FRA to have the value 1 in each time bin and 0 with probability 1 − FRA, (0 ≤ FRA ≤ 1).  The number of bins in a spike train is set in the model to equal the duration of each stimulus. For example, if a stimulus appears within the visual field for 100ms then the neurons whose receptive fields correspond to that stimulus will generate spike trains that contain 100 time bins.

The firing rates of the input neurons are established using the saliency algorithm of Walther and Koch (2006). The overall saliency at each location in the visual field results from the integration of information across individual feature maps. The output of the algorithm is the final “saliency map” that is represented by a grayscale image that indicates the computed saliency value of each pixel. This final saliency map is used to generate the initial firing rates of the input neurons according to Eq.B1 (see also Figure 3):

$FR_{si}=\alpha(Max(Pj))+\beta(\sum_{j=1}^{n} Pj)$ Eq.B1

In Eq.B1, *FRsi* represents the firing rate of each of the 12 input neurons that correspond to the receptive field of the neurons that correspond to stimulus *Si.* *Max (Pj)* $\left( \mathrm{Max}\left( \mathrm{Pj} \right) \right)$is the maximum value among all the pixels that correspond to stimulus *Si*, and $\left( \sum_{j=1}^{n} \mathrm{Pj} \right)$ is the total summation of the n pixel values (*Pj*) that correspond to stimulus *Si*. The terms *α* and *β* are weighting constants. The maximum pixel value for each stimulus reflects the general saliency of the stimulus while the summation value is used to incorporate the influence of stimulus size, as the model employs a fixed number of 12 neurons to encode incoming stimuli regardless of their size (Figure B1).


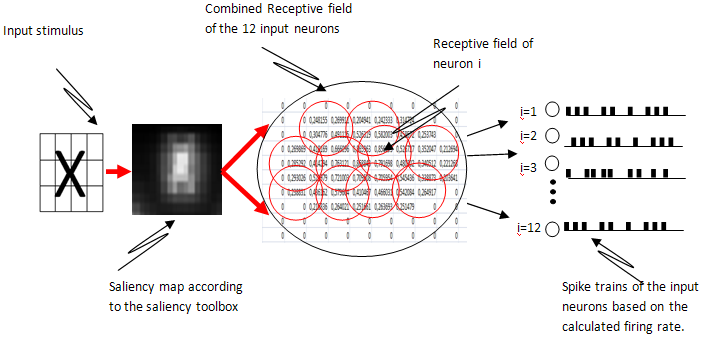


*Figure B1*. Establishing initial firing rates using a saliency map algorithm.

**Appendix C**

Figure C1 presents a graphical example of the effect of spatial cueing as implemented in the model. The visual stimuli in the example are the letters A, which is cued by an arrow, and the letter O. In the example three layers of neurons are shown. In the first layer the neural activity that corresponds to each of the two letters is similar due to their similar saliency values. The effect of spatial cueing can be observed in layers 2 and 3, where high frequency spike trains interact with the network of CD nodes that are linked to the neurons whose receptive field coincides with letter A. This interaction results in higher frequency neural activity in layer 2 for the neurons that correspond to letter A than those that correspond to letter O. This, in turn, leads to stronger inhibition towards the letter O from the pool of inhibitory neurons linked to the neural activity associated with letter A than the other way around (as seen in the third layer of neurons in the example).


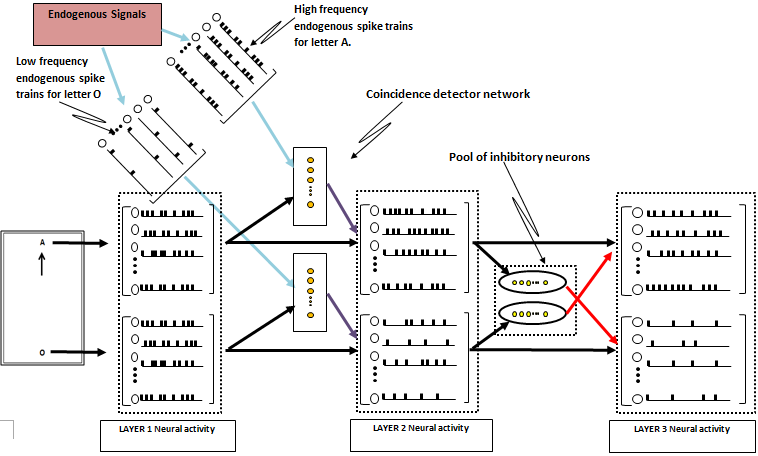


*Figure C1*. The effects of spatial priming on the neural activity of stimuli

The frequency of the spatial top-down signals in the model depends linearly on the number of spatial locations that are primed either by spatial cues or by task instructions. For example, when a spatial cue primes the location of an upcoming stimulus, then spatial top-down signals with a spike train frequency = *f* will interact with the input neurons whose receptive fields correspond to that specific location of the visual image. In the case where multiple cues prime the locations of several stimuli, the activity of the neurons whose receptive fields correspond to the cued locations is influenced by spatial top-down signals with spike train frequency equal to $f/N$ , where *N* is the number of cued locations.

**Appendix D**

**The temporal filter**

The temporal filter mechanism used in the model is inspired by Crick and Koch’s (1990) suggestion that the selection of stimuli could be made on the basis of synchrony across neurons. Based on neurophysiological findings that visual stimuli can elicit synchronized activity in the visual cortex, Crick and Koch (1990) suggested that a prerequisite for the presence of neural synchronization during attention tasks might be the appearance of synchronous impulses in selected neuronal populations. They proposed that visual selective attention causes changes to the temporal structure of the neural spike trains that represent the information to be selected, facilitating thus the transfer of the encoded information to WM. In a comprehensive review, Womelsdorf and Fries (2007) present evidence from many studies showing that attention modulates the firing rates of neurons that represent the attended stimulus features causing synchronization. For example, Bichot, Rossi and Desimone (2005) recorded the neuronal spiking responses and Local Field Potentials in the visual area V4 of macaque monkeys and demonstrated that the allocation of attention towards a particular feature resulted in synchronized responses of sensory neurons tuned to that feature. Bichot et al. (2005) suggested that the increase of synchronization and firing rates in V4 neuronal spiking responses (during feature selective attention tasks) is correlated with the similarity between the features of the attended stimulus and the task’s feature preferences.

The temporal filter module encapsulates an algorithmic function with its inputs being: i) the predefined probability of resemblance $P(res)$ for each incoming stimulus (i.e. for stimulus $s_{i}$ that would be ${P(res)}_{s_{i}}$), ii) the spike sequence of the previous layer neurons, and iii) the spike train that corresponds to a semantic representation of a specific target. The output of the algorithm is a new spike train with the same frequency of response but with an adjustment on the timing of spike appearance. The probability of resemblance is computed using a value-based matrix that is set according to how similar the feature characteristics of a given stimulus are (e.g., the orientation of lines, the number of line crossings, the presence of curves) to those of the target. For example, consider the case that the target is the letter X, and the letters O and A appear in the visual field. According to the value-based matrix, the letter A will be assigned a higher probability of resemblance than the letter O, as its features (e.g., diagonal lines and crossing points) are more similar to those of the target. The higher probability of resemblance increases the probability of modifying the temporal pattern of the spike trains that correspond to the letter A so that they are more similar to those in the target representation maintained in the Endogenous Module.

The temporal filtering mechanism is presented in Figure D1 where $y_{end}$ denotes the endogenous spike trains and ${y_{FS}}_{Si}$the spike trains of the neurons that correspond to a visual stimulus Si. Specifically, a random number is generated which the algorithm compares to the probability of resemblance that corresponds to the stimulus Si (Figure D1). If the random number is smaller or equal to the specific probability, the time bin of the incoming spike $t_{k_{\mathrm{FS}_{s_{i}}}}$will change and will appear in the closest time bin that a spike appears within the endogenous spike train time sequence $t_{k_{\mathrm{end}}}$. In case the random number is greater than the ${P_{\mathrm{res}}}_{s_{i}}$, the appearance of the incoming spike will remain in its original time bin. In this manner, it is possible to “re-organize” the temporal pattern of an incoming stimulus without interfering with its firing rate, since the total number of spikes remains the same.


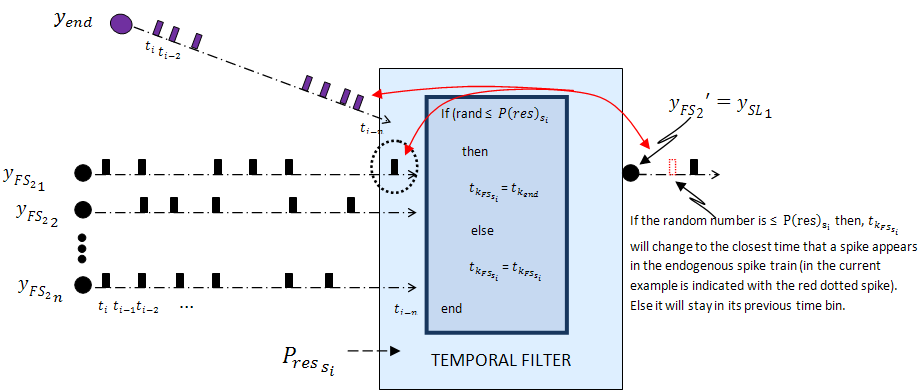


*Figure D1*. The temporal filter mechanism

**Appendix E**

**The Control Module**

In the Control Module, each CD neuron has three neurons connected to its input: two inputs randomly selected from the total number of neurons corresponding to the neural activity of an incoming visual stimulus and one input from the target representations. The response of the CD nodes is explicitly dependent on the number of action potentials that arrive simultaneously at their inputs. In the current implementation, the threshold has been set to θ =2. That is, at least two spikes of the three inputs on each CD node must arrive synchronously for the node to fire (see Figure E1). At the same time, an increase in the degree of correlation will generate increase in the synchronization of neural activity in the spiking neural network of the second stage of processing. Thus, a strong correlation between an incoming stimulus and the endogenous signals will result in an increase of the firing rate and a gradual increase of synchronous firing by the CD nodes.


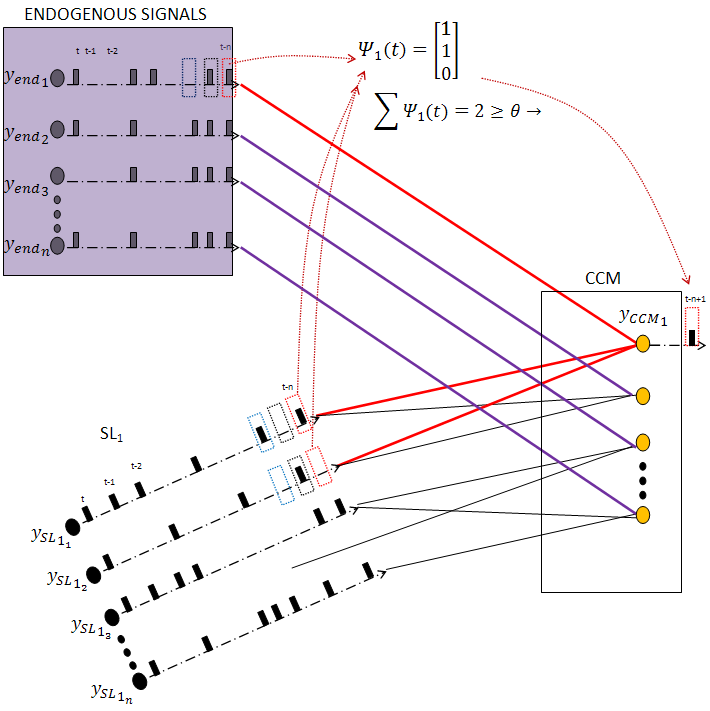


*Figure E1*. The Correlation Control mechanism

In Figure E1 $\Psi_{1}(t)$ is a binary vector that denotes the states of the spike trains from the endogenous goals $y_{\mathrm{end}_{i}}(t)$ combined with the states from two neurons of the incoming visual stimulus during the second stage of processing ($y_{\mathrm{SL}_{1_{1}}+}y_{\mathrm{SL}_{1_{2}}}$)
